# Early-Life Interpersonal and Affective Risk Factors for Pathological Gaming

**DOI:** 10.3389/fpsyt.2020.00423

**Published:** 2020-05-15

**Authors:** Silvia Bussone, Cristina Trentini, Renata Tambelli, Valeria Carola

**Affiliations:** ^1^Department of Dynamic and Clinical Psychology, Sapienza University of Rome, Rome, Italy; ^2^Department of Experimental Neuroscience, Santa Lucia Foundation (IRCCS), Rome, Italy

**Keywords:** internet gaming disorder, early-life stress, family functioning, attachment, childhood maltreatment, bullying

## Abstract

Internet gaming is among the most popular entertainment options, worldwide; however, a considerable proportion of gamers show symptoms of pathological gaming. *Internet gaming disorder* (IGD) has been proposed to describe a behavioral addiction, which shares many similarities, both physical and psychological, with substance use disorder. Environmental factors, such as interpersonal and relationship dynamics during childhood and adolescence, have been suggested to modulate the onset and trajectories of IGD. However, studies exploring the contributions of dysfunctional family environments to the development of IGD remain limited. This minireview aims to offer an overview of the current knowledge regarding the impacts of early-life interpersonal and relationship dynamics on the development of IGD and to provide a snapshot of the current state of the literature in this field. Specifically, it underlines the modulatory role of early-life relational factors such as a) family function, b) parent-child relationships, c) childhood maltreatment, and d) bullying and cyberbullying on the development of IGD. Consistent with this evidence, therapeutic interventions that aim to “restructure” the emotional ties and familiar dynamics that are known to be associated with dysfunctional behaviors and feelings, and likely promote pathological gaming, are recognized as the most successful clinical therapeutic approaches for IGD.

## Introduction

The video game sector is among the most popular entertainment options worldwide (1). In a healthy context, gaming can have many positive impacts, including educational, social, and therapeutic functions (2), and most gamers are recreational game players, who do not experience cravings or other symptoms that are typical of addiction (3).

However, the detrimental effects of gaming have been described for a minority of gamers (4). Between 0.7% and 15% of gamers show symptoms of problematic gaming, with the highest rates identified in males (5). The harmful consequences of video game use include physical and psychological disorders, social deficits, and/or poor academic performance (6).

### Pathological Gaming

Historically, the concept of pathological gaming as an addictive disorder falls within the field of pathological gambling and substance use disorder (7). The first reports evaluating gaming problems employed assessment instruments that were adapted from questionnaires commonly used during pathological gambling research (8).

The latest edition of the Diagnostic and Statistical Manual of Mental Disorders (9) included diagnostic criteria for internet gaming disorder (IGD) in the appendix but also described IGD as a condition that warrants further investigation (10). IGD is a new behavioral addiction, defined as the “*recurrent and persistent use of internet games leading to significant psychosocial functional impairment*” (9). The DSM-5 diagnostic criteria included: preoccupation or obsession, withdrawal, tolerance, loss of control, loss of interest, continued overuse, deceiving, escape of negative feelings, and functional impairment. At least five of these nine criteria must be met for a period of at least 12 months to qualify an individual for an IGD diagnosis (9). IGD was also included in the latest revision of the International Classification of Diseases (ICD-11, 11), as “gaming disorder” (11), defined as a *“recurrent gaming behavior pattern that includes both online and offline gaming”* (11).

Although observable differences exist between the ICD and DSM systems for the clinical description of IGD, both systems have established that IGD shares common characteristics with other addictive disorders, such as drug abuse (12) and pathological gambling (e.g., the prioritization of gaming over other activities, loss of control, and functional impairment) (13), and both define IGD as a pattern of repetitive or persistent (pervasive) gaming behavior (9, 11). Controversies continue to exist regarding the IGD definition, and whether IGD should be classified as a compulsion, an impulse control disorder, or a behavioral addiction remains unclear (14). However, the formal inclusion of pathological gaming as a behavioral addiction has received strong support from the fields of clinical psychology and psychiatry and the public health system (15).

### Psychological and Behavioral Changes Observed During IGD and the Neural Correlates Associated With Those Changes

A series of studies investigating the addictive potentials of gaming tools identified several factors that may modulate IGD susceptibility during adolescence (16), among which *achievement*, *socializing*, and *immersion* have been identified as being the most significant (17). Game advancement, through leveling up, and earning the admiration of the gaming community can enhance the sense of *achievement* experienced by gamers and encourage gamers to dedicate more effort to gameplay (14, 17). A further incentive for pathological gamers, especially those who experience loneliness in the real world, is game-based *socializing*, which presents the abilities to chat, work in teams, and make new friends through the game (14). Finally, when gamers experience negative moods or thoughts, including fear, anxiety, and depression (18), game players may be incentivized to escape real life and experience *immersion* in the gaming world (14).

Several studies have reported an association between IGD and low self-esteem, which suggested that players may depend on gaming to acquire self-esteem, to compensate for weak self-images by displaying game mastery, to escaping reality, to overcome social tribulations, or to fulfil the needs of social reinforcement (19). Moreover, evidence has suggested a negative association between self-efficacy (the absolute trust in one’s abilities to produce outcomes) and IGD, which suggested that addicted gamers may harbor impaired self-concepts (20).

Alterations in several brain functions have been detected in IGD, including alterations in reward and motivational processes (21, 22), executive function, and cognitive control (21). The impacts of gaming on reward and motivational systems are supported by evidence showing the increased sensitivity to rewards among IGD individuals (23), which results in increased functional brain responses to gaming (23, 24). Moreover, recent neuroimaging studies that have examined IGD have confirmed the presence of dysfunctions in the motivational and reward system, demonstrating altered functional connectivity in both the ventral tegmental area-nucleus accumbens pathway and the ventral tegmental area-medial orbitofrontal cortex pathway (which are relevant for dopamine reward signals and salience attribution respectively) (22).

The evidence that the motivational and reward systems are involved in behavioral addictions (e.g., IGD) as well as in substance use disorder has inspired several theories suggesting that these clinical manifestations may result from the same underlying vulnerability, associated with several neurobiological, genetic, psychological, and social risk factors (25), rather than a consequence of exposure to a specific substance or behavior (26). In light of these theories, attempts have been recently made to identify individual’s profiles, combining family environment, personality, and mental health factors, associated with vulnerability to addictions. The seven identified profiles are differentially associated with behavioral addictions and substance use disorder, with some profiles that are linked to both behavioral addictions and substance use disorder, whereas others that are characterized by more specificity for only one addiction typology (27).

Moreover, investigations have documented significant alterations in executive brain functions among IGD individuals, characterized by low executive control (e.g., response inhibition failure, impaired error monitoring, and high impulsivity), low cognitive flexibility, and enhanced disadvantageous decision-making (23, 28). Functional and structural neuroimaging studies performed in IGD individuals have detected abnormalities in brain regions relevant to brain executive and cognitive control, such as the superior temporal gyrus, anterior cingulate cortex, dorsolateral prefrontal cortex, and orbitofrontal cortex (29, 30).

### Early-Life Interpersonal and Affective Risk Factors for Pathological Gaming

**Figure 1:**
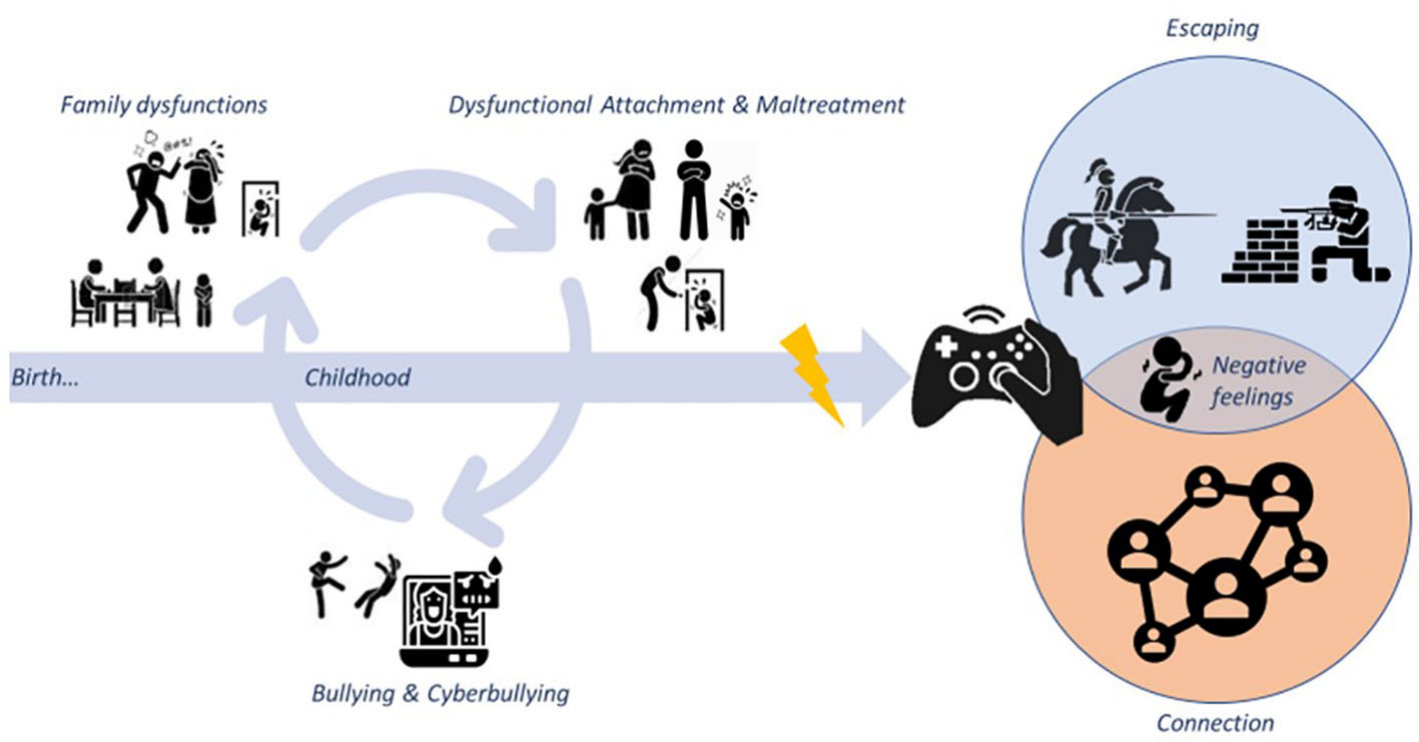
Interpersonal and relationship dynamics during childhood and adolescence have been suggested to modulate the onset and trajectories of IGD. Among social risk factors, family malfunction, dysfunctional parent-child relationship, maltreatment, bullying and cyberbullying have a relevant role in the etiopathogenesis of IGD. Those factors could prompt teenagers to “escape” the real world by fleeing into the cybernetic world, and gaming could represent a coping strategy that is implemented by the child/adolescent to escape from unpleasant feelings raised in family and social contexts. IGD, Internet gaming disorder.

Understanding the risk factors associated with IGD is necessary to correctly predict, diagnose, and treat this emerging disorder. Environmental factors, such as cultural, socioeconomic, parental, and external stressors (31), have been suggested to act as modulators of the onset and trajectory of IGD. Interpersonal dynamics and relationships have been shown to be pivotal for the development of behavioral addictions [(32), see **Figure 1**]. Early-life family-related variables have been reported as significantly affecting the likelihood of an adolescent becoming a problem gamer. Among them parental influence on gaming modalities (e.g., supervision of gaming), family environment and functioning (e.g., domestic composition), parent–child interactions (e.g., attachment relationships and conflict), and parent status (e.g., socioeconomic status and psychological health) seem to play relevant role (33). Poor family relationships and family relational trauma may lead an adolescent to seek out social engagement in gaming activities and use online activities as a coping mechanism (34). Notably, pathological and excessive gaming in adolescents can worse family functioning and may displace opportunities for family interaction (35). However, despite a wide range of studies has examined and described the relationship between early-life dysfunctional socio-familiar dynamics and IGD development, this association needs to be further investigated.

This minireview aims to offer an overview of early-life interpersonal factors that are relevant for the development of IGD, by specifically focusing on direct and indirect influences of a) family functioning, b) parent-child relationships, c) childhood maltreatment, and d) bullying and cyberbullying. It collects evidence that demonstrates the decisive role played by the early-life family and social environment in the development and maintenance of IGD. A critical examination of this literature may help psychologists and psychiatrists to pay greater attention to early-life socio-relational dysfunctions, targeting these dysfunctions in therapeutic contexts, with a consequent increase in the success rate of IGD clinical treatment.

## Methodology

The authors conducted a literature search of available sources describing the issue of IGD, with specific focus on interpersonal issues. Research studies were selected on the basis of research topics (such as the definition of internet gaming, gaming and familiar functioning, gaming and attachment styles, gaming and childhood maltreatment, gaming and bullying and cyberbullying) found in the world’s acknowledged databases, such PubMed and Google Scholar, from the period of 2000 up to date. As narrative minireview, research reports, case reports, systematic reviews and meta-analyses were considered for retrieving data. These articles were classified according to their relevance to the minireview topic and included if they substantially contribute to the narrative.

## Results

### Family Functioning

Among social risk factors, the family environment appears to play a relevant role in the promotion of pathological gaming (36). Although research on the role of family functioning in IGD development is currently ongoing, global family functioning appears to affect the developmental trajectories of IGD (33, 36). Evidence suggests that the regulation and monitoring of gaming among children represents an effective strategy for preventing the onset of IGD (37). Families that regulate gaming may be more likely to direct an adolescent’s attention toward other recreational activities, rather than video games (38). Good parental monitoring is also associated with active parental participation in childcare (37). Additionally, psychosocial factors, such as low parental education levels (39), parental divorce or separation (40), and single-parent families (41), have emerged as predictors of IGD symptoms, which may develop as a result of reduced child social competencies.

Problematic gamers have been reported to experience significantly more family conflicts, more negative perceptions of the family environment, and worse family relationships than non-pathological gamers, whereas non-pathological gamers have significantly better family cohesion than pathological gamers (36). Moreover, children in families that are characterized by high levels of conflict or discord appear to exhibit more frequent problematic gaming, as do children from single-parent families (41).

Research on addictive behaviors and family functioning during adolescence has also found that a low level of family adaptability (reflecting a family’s ability to adapt its power structure, role relationships, and rules to respond to situational or developmental needs) is a relevant factor that can predict addictive behaviors, whereas low cohesion (reflecting the emotional bonds that exist between the members of a family) appears to predict only the hedonistic dimensions associated with addictive behaviors (42). Pathological gaming can activate a vicious circle, in which gaming has the potential to worsen already poor family functioning, which, in turn, can worsen pathological gaming (43). IGD can also disrupt family functioning, causing problems in daily life and disrupting the relationships between gamers and other family members. In parallel, family “dysfunctionality” could prompt teenagers to “escape” the real world by fleeing into the cybernetic world (43), and gaming could represent a coping strategy that is implemented by the child/adolescent to escape from unpleasant family conditions (33) or to avoid feelings, such as sadness or anger, that are induced by adverse family functioning [(33); **Table 1**].

**Table 1:** Studies of early-life interpersonal and affective risk factors for IGD.

Study	Article type	Sample age	Sample size	Main findings
**Family functioning**				
*36*	Research report	13.2	434	Parental monitoring and family functioning are associated with IGD.
*33*	Systematic review	Under 18	14 studies	Poor family relationship is associated with IGD. A positive father–child relationship may be protective against IGD.
*37*	Research report	8–12	5,864	The monitoring of gaming among children represents an effective strategy for IGD prevention.
*38*	Research report	10–15	1,490	Positive parent–child relationship (e.g., higher parental monitoring and better father–child relationship) prevent IGD in adolescents.
*39*	Research report	8–10	740	Low parental education and less child word comprehension predict IGD, seemingly through reduced child social competencies.
*40*	Research report	13–18	1,231	IGD and substance dependence can be described by similar criteria. IGD displays connections with specific psychosocial (e.g., parental divorce), psychopathological, and motivational factors.
*41*	Research report	10–15	1,217	Growing up in a single-parent family and problematic use of videogames in childhood are risk factors for IGD.
*42*	Research report	17.59	252	Family adaptability is associated with addictive behavior development. Low family cohesion predicts only the hedonistic dimensions associated with addiction.
*43*	Research report	Adolescence	903	Parenting attitudes, family cohesion, and exposure to family violence could prompt the teenager to "escape" the real world feeling into videogames.
**Parent–child relationships**				
*44*	Research report	Under 18	225	The quality of parent–child interaction is associated with IGD. Parental rejection predicts IGD, through the mediation of CSE.
*45*	Research report	11–13	2,974	Longitudinal data analysis identifies negative parent–child relationships as risk factors for IGD. A moderating effect of child/adolescent gender is also observed.
*46*	Research report	10–18	2,527	IGD individuals have a worse perception of their family environment. Gamers have a better relationship with their mother than their father.
*48*	Research report	Adulthood	141	Anxious attachment and psychopathology interaction predicts problematic internet use.
*49*	Research report	20–30	200	Anxious attachment has a severe impact on smartphone addiction. This relation is mediated by loneliness and depression.
*50*	Research report	17–49	207	Anxious and avoidant attachment predicts social media addiction.
*51*	Research report	13–21	472	Emotional dysregulation predicts both substance use disorder and behavioral addictions. Attachment dysfunction predicts only behavioral addictions.
*52*	Research report	18–51	252	Gamers with anxious/avoidant attachment develop pathological gaming more than gamers with secure attachment. All gamers played to immerse themselves into a fantastic world.
*54*	Research report	25	337	The relationship between attachment (anxious/avoidant attachment) and IGD is mediated by stressful events.
**Childhood maltreatment**				
*59*	Research report	13–38	242	Exposure to emotional abuse and/or neglect is a risk factor for IGD.
*60*	Research report	12–16	1,868	Adolescents experiencing school bullying and family maltreatment show problematic gaming. Problematic gaming is associated with psychiatric symptoms.
*63*	Case report	13 and 15	2	In two clinical cases (with respectively, externalizing and internalizing symptoms), attachment plays a role in IGD onset and maintaining, and gaming represents a maladaptive self-regulatory strategy.
*64*	Narrative review			Compensatory internet use model: this model assumes that people go online to escape real-life stressful issues and alleviate dysphoric moods.
*18*	Research report	17–24	174	A positive correlation is observed between time spent playing video games and social anxiety, whereas a negative correlation is observed with social contact quality.
*65*	Case report	23 and 38	2	Technological addictions in traumatized subjects promote emotional deactivation and detachment from overwhelming psychological states, caused by early-life stress.
*66*	Research report	12–18	31	IGD individuals report family dysfunctions and maltreatment. Exposure to PIPATIC group therapy significantly reduces IGD symptoms.
*67*	Research report	17–59	103	Maladaptive personality traits in combination with gaming-related positive and negative expectancies are important factors for IGD development.
**Bullying and cyberbullying**				
*68*	Research report	12–19	823	Adolescents showing cognitive and behavioral avoidance, as a coping strategy, have a greater risk of developing IGD.
*69*	Research report	14–15	2,008	Mobile game bullying is relatively common, although severe bullying is rarely reported. Male players from a minor ethnicity and players with conduct problems are more likely to report victimization.
*70*	Research report	18–25	344	Cyberbullying perpetrators show problematic social media use, dissociative experiences, cluster B traits, depression, childhood emotional trauma, and low self-esteem.
*73*	Systematic review	Childhood, adolescence, adulthood	37 studies	In adolescents, IGD is associated with problems with peers (high prevalence of being bullied, bullying others and having friends addicted to video games), with low educational and career attainment (low school grades, skipped school classes, and truancy), and with low social skills, competence, and integration.

PIPATIC, Programa Individualizado Psicoterapéutico para la Adicción a las Tecnologías de la información y la comunicación; IGD, internet gaming disorder; CSE, core self-evaluation.

### Parent-Child Relationships

Instability and/or dysfunction associated with parent-child interactions may contribute to the development of IGD (44). Longitudinal studies have reported that positive parent-child relationships contribute to the prevention of IGD development and reduce existing symptoms associated with problematic gaming (45). Problematic gamers appear to have worse relationships with their parents than normal gamers but often report better relationships with their mothers than with their fathers (46).

A link between early-life attachment relationships and the susceptibility to substance use disorder has been consistently reported (47), and recently, a similar link has been described between attachment and technological addictions, such as smartphone, internet, and social media addictions (48–50).

Although studies exploring the contributions made by “dysfunctional” attachments to IGD development remain limited (51), evidence suggests that gamers who are characterized by *insecure attachment* (those who are *anxious* and *avoidant*) display more problematic gaming behaviors than those with a *secure* attachment style (52). *Anxiously* attached individuals exhibit an exaggerated need for interpersonal closeness and support, due to their perceived inability to handle stress autonomously (53). Among these individuals, pathological gaming may be driven by the potential of games to quell their distress and to fulfil their closeness needs (19). In contrast, *avoidant* individuals suppress their needs for interpersonal intimacy, to prevent frustrations associated with social rejection (53). Among these individuals, pathological gaming may be driven by the potential of games to suppress their negative emotions and to deactivate their needs for attachment relationships (54).

The direct and indirect impacts of childhood parental acceptance and rejection [PAR, (55)] on IGD development have been investigated. PAR does not appear to be directly associated with addictive gaming behaviors; instead, the relationship between PAR and IGD is mediated by PAR-induced changes in core self-evaluations (CSEs), which are personality constructs that represent the fundamental appraisals/judgments that individuals apply to themselves, other people, and the world (56). The more rejection an individual experiences at an early age, the more likely that individual is to develop low CSEs (i.e., low self-esteem and self-efficacy), which may result in the individual becoming more prone to the development of pathological gaming behavior (see *Pathological Gaming*). New technologies may offer environments for adolescents to develop their self-esteem and identity (51), and non-substance-related addictions may be viewed as being associated with the need for relationship satisfaction [(51), **Table 1**].

### Childhood Maltreatment

Family dysfunction is often associated with child maltreatment, which can describe sexual, emotional, or physical abuse and emotional and physical neglect (57). Childhood maltreatment is known to produce dramatic negative consequences, such as feelings of shame and guilt, poor social relationships and psychological functioning, reduced self-esteem, and increased engagement in risky and impulsive behaviors (58). Maltreatment can be considered to represent the failure of caregivers to meet the basic needs of children (i.e., love, belonging, nurturing, and support (59). Experiences of victimization, such as family maltreatment, are frequently reported to be associated with IGD susceptibility in children and adolescents (60). Exposure to emotionally traumatic events during childhood has been demonstrated to play a pivotal role in the onset and maintenance of addictive disorders, especially the addictive use of the internet and online games (59, 61). Additionally, parental physical abuse has been reported to be associated with problematic gaming in adolescents (60). Children who are exposed to abusive nurturing practices are likely to develop both internalizing and externalizing (62) symptoms which, in turn, have been demonstrated to be susceptibility factors for IGD (63).

In accordance with “the Compensatory Internet Use (CIU) model” (64), the addictive use of the internet and online games appears to be used to functionally compensate for unmet social and emotional needs (e.g., achievement and social affiliation) or to cope with psychological suffering, such as depression and anxiety (18), among early-life-traumatized individuals (64). The CIU model conceptualizes IGD as a dysfunctional coping strategy, used to counteract emotional distress and psychopathological symptoms consequent to adverse early-life events (59, 61, 64).

Excessive video game playing in traumatized subjects appears to play a functional role that promotes emotional deactivation and detachment from overwhelming psychological states, caused by early-life-traumatic experiences (65). In a chaotic, rejecting, or threatening family environment, gaming may be the only viable coping strategy (33). In support of this hypothesis, many individuals who have experienced stressful life events have developed IGD (66). Gamers who are prone to experiencing psychological stress or discomfort during everyday life, due to maladaptive personality traits, are the most likely to develop problematic gaming behaviors [(67) **Table 1**].

### Bullying and Cyberbullying

Psychological distress that is associated with victimization, due to maltreatment, has been positively correlated with problematic gaming, through the mediating effects of gaming drivers, such as escape and competition (68). Thus, adolescents who experience bullying at school, mistreatment at home, and/or who experience anxious or depressed feelings, due to their living situations, may view video/online gaming as a method for escaping or coping with the real world (60).

Online/video games provide necessary opportunities for competitive and social play and potentially promote the building of new social relationships. However, games may also provide an avenue for negative social experiences, such as bullying or trolling behaviors (69).

Bullying in online games appears to be a relatively common phenomenon, and those adolescents who already struggle with psychosocial difficulties in the real world are the most likely to either report victimization (69) or to perpetrate bullying in virtual contexts (70). Children who experience parental maltreatment are considered to be more likely to reproduce victimization and violence in extrafamilial relationships (71). Under dysfunctional family conditions, children internalize aggressive and abusive experiences as relationship patterns that must be endured or reproduced in all other relationships (72).

Adolescents with problematic gaming behaviors who are victimized by bullying and cyberbullying often present with concomitant school problems and worse social competencies than peers with non-problematic gaming behaviors [(73), **Table 1**].

## Discussion

Overall, the studies presented here indicate how pathological gaming is often conceptualized as a method for escaping or coping with the real world when low self-esteem and low self-efficacy are pervasive in an adolescent’s life (74). Pathological gaming has been considered to be a strategy that individuals use to compensate for weak self-images because games facilitate the display of mastery (75) and allow individuals to acquire more confidence in their own abilities (76).

Research has extensively investigated the factors that modulate susceptibility to pathological gaming, including *achievement*, *socializing*, and *immersion* (17), which fulfil the needs of individuals to receive admiration from others (14, 17), to experience a sense of companionship (14), and to cope with feelings of stress, anxiety, and depression (14, 18, 77), respectively. These aspects of gaming are particularly relevant when we consider the complex developmental tasks that individuals experience during adolescence, especially developing a sense of identity (78) and engaging in peer relationships (79). To overcome these developmental demands, adolescents must experience high levels of support and intimacy within their families (80). Moreover, family functioning, the quality of the parent-child relationships (e.g., attachment), and early traumatic experiences appear to be critical risk factors for the development of pathological gaming behaviors (36, 44, 60).

Recently an association between IGD and Hikikomori (81), a phenomenon featured of prolonged social withdrawal, has been described in adolescents (82). Interestingly, several studies investigating early-life interpersonal factors relevant for Hikikomori development (82, 83), reported factors similar to the ones described for IGD in this minireview. Hikikomori individuals indeed report family profiles characterized by single-parenthood and failure to fulfill the individual’s needs for affection. Nevertheless, to verify if IGD and Hikikomori share similarities in the psychosocial functional impairment and its origin, further studies are absolutely needed.

Among the clinical therapeutic approaches that are currently used to treat IGD, the most successful approaches apply protocols that combine an *intrapersonal* approach with an *interpersonal* approach (66). The first approach [usually a cognitive behavioral therapeutic (CBT) approach, (13)] aims to achieve the cognitive restructuring of CSEs, which results in improved self-esteem and social skills. The goal of CBT, here, is the regulation of aspects that IGD shares with substance use disorder, such as stimulus control, self-monitoring strategies, problem-solving related to addiction, and withdrawal regulation techniques with exposure (84). The implementation of an *intrapersonal* clinical approach has been shown to be effective when combined with an *interpersonal* approach (usually a systemic orientation approach), in which proximal psychosocial relationships (e.g., family and parent-child relationships) and familiar dynamics are “restructured,” to reduce the dysfunctional behaviors and feelings that promote pathological gaming (85).

Similar to other psychological domains, research on the effectiveness of psychodynamic treatments for IGD in adolescents is scanty when compared with research examining the thorough application of the CBT approach. This general disparity in research focus may be due to the specificity of the therapist-patient relationship during psychodynamic treatment, which includes specific dimensions (such as transference and counter-transference) and psychodynamic phenomena (e.g., ego and defense mechanisms) that are difficult to operationalize into psychological variables for experimental testing (86). However, a single-case study has reported that psychodynamic treatment was effective for treating pathological gaming, improving self-esteem, and reducing problematic relationships with peers and aggressive tendencies towards family (87).

This review has limitations that warrant acknowledgment. Due to its narrative rather than systematic nature, this minireview may not include several recent reports and articles that were not considered contributing to its narrative. For a more detailed and broader analysis of the recent literature on this topic, it would be adequate to refer to reviews that have used a systematic approach [e.g., (33)]. The long-term relevance of early-life interpersonal and affective factors on IGD has never been assessed because of the lack of longitudinal studies investigating this issue.

Despite the reported constraints, this minireview can be relevant for prevention and clinical practice. The interpersonal factors associated with IGD here described may help educators and psychologists in recognizing adolescents at risk for developing this disorder. Preventive and educational programs should be then aimed at informing the adolescent at risk and his family about the harmful consequences that excessive use of video game may have on the individual’s emotional and behavioral functioning.

The findings summarized in this review seem to support the utility of a clinical approach that includes the entire “IGD family” (adolescent with his parents) in the therapeutic process with the aim of improving communicative competences and cohesion in the family.

## Conclusion

Taken together, the current research indicates the need to focus on socio-relational dimensions—particularly those referred to the family environment—that have been proven to have a crucial role in the development as well as maintenance of IGD in adolescence. To obtain positive outcomes, both prevention programs and therapeutic treatments cannot avoid addressing these specific dimensions. The results summarized this review may provide a useful guide for further empirical research that, beyond addressing early interpersonal risk factors for IGD, could investigate the intra- and interpersonal protective factors that mitigate the risk for the development of IGD during adolescence.

## Author Contributions

SB, CT, RT, and VC wrote the manuscript. SB designed the figure.

## Conflict of Interest

The authors declare that the research was conducted in the absence of any commercial or financial relationships that could be construed as a potential conflict of interest.

The reviewer GK declared a shared affiliation, with no collaboration, with the authors to the handling editor at the time of the review.
